# Effect of Polystyrene Synthesis Method on Water Sorption and Glass Transition

**DOI:** 10.3390/membranes12111059

**Published:** 2022-10-28

**Authors:** Daniel T. Hallinan, Matteo Minelli, Onyekachi Oparaji, Andrea Sardano, Oluwagbenga Iyiola, Armando R. Garcia, Daniel J. Burnett

**Affiliations:** 1 Department of Chemical and Biomedical Engineering, Florida A&M University–Florida State University College of Engineering, 2525 Pottsdamer Street, Tallahassee, FL 32310, USA; 2 Aero-Propulsion, Mechatronics, and Energy (AME) Center, Florida A&M University–Florida State University College of Engineering, 2003 Levy Avenue, Tallahassee, FL 32310, USA; 3 Department of Civil, Chemical, Environmental and Materials Engineering (DICAM)—Alma Mater Studiorum, University of Bologna, Via Terracini 28, Bologna 40131, Italy; 4 Interdepartmental Center for Industrial Research in Advanced Mechanical Engineering Applications and Materials Technology (MAM)—Alma Mater Studiorum, Viale del Risorgimento 2, 40136 Bologna, Italy; 5 Surface Measurement Systems, 2125 28th Street SW, Suite 1, Allentown, PA 18103, USA

**Keywords:** polystyrene, radical, anionic, humidity, plasticize, *T_g_* depression, poor solvent, water, uptake, temperature, modulus

## Abstract

Commodity PS is synthesized via free radical polymerization, whereas PS in block copolymers (BCPs) is typically synthesized via living anionic polymerization. The purpose of this work is to investigate how the synthesis method impacts important properties such as water sorption and glass transition temperature (*T_g_*). Water sorption is important because the performance of nanostructured polymer membranes in various applications is known to be affected by environmental conditions such as humidity. *T_g_* is important because it dictates processing conditions, both for commodity PS as well as BCPs such as thermoplastic elastomers. Water sorption in commercial PS was found to be 0.5 mg_water_/g_polymer_ at the highest humidities investigated (about 80%), in agreement with literature. On the other hand, syndiotactic PS synthesized anionically at low temperature absorbed more water, up to 1.5 mg_water_/g_polymer_, due to higher free volume. The greatest impact on water sorption was due to addition of hydrophilic hydroxyl chain ends to atactic PS, which resulted in water sorption of up to 2.3 mg_water_/g_polymer_. In addition to measuring water sorption and dry *T_g_* separately, the impact of relative humidity on PS *T_g_* was examined. Combined differential scanning calorimetry and dynamic mechanical analysis show that on going from the dry state to high humidity, the *T_g_* of PS decreases by 5 °C. Moreover, the tensile storage modulus of PS decreases from 1.58 GPa at 0% RH to 0.53 GPa at 40% RH. In addition to the practical relevance of this study, this report fills a gap in experimental literature by using a poor solvent system, PS/water, to examine plasticization in the pure polymer limit.

## 1. Introduction

Polystyrene (PS) is a widely studied and broadly used thermoplastic. In 2015, it accounted for over 8% of the demand for standard thermoplastics with over 18 million tons produced [1]. It is commonly used in food packaging and insulation [2]. It is a particularly interesting polymer due to its ability to be incorporated into block copolymers (BCPs) for more specialized applications, such as membrane-based separations [3,4], batteries [5,6,7,8], and nanolithography [9,10,11,12], Some of these applications require mechanically strong membranes, which can be achieved by combining glassy PS with a rubbery polymer (often as a BCP). Many applications require macroscopic processing or mesoscopic self-assembly. Both of these can be achieved at temperatures above the glass transition temperature (*T_g_*) of PS but well below the degradation temperature. This ease of processability into a variety of forms is the single most important reason for the ubiquity of PS.

In addition to the obvious interaction of PS foams and films with water vapor in the atmosphere, water interaction with PS is relevant in numerous other applications. Despite the general assumption that PS is hydrophobic and absorbs negligible amounts of water, diffusion of water in PS has been found to be significant, for example, in expanding PS with water from polymerized emulsions [13,14]. Other situations of technical interest include chemical recycling of polystyrene [15,16] to address microplastic waste issues [17], as well as filtration and separation processes [18,19,20]. Interesting but rather obscure situations in which water and PS are closely interacting include coal liquefaction [21] and water-based lubricants [22]. A timely use of PS is for protecting perovskite-based photovoltaics from water based degradation [23]. Perhaps most importantly, PS is widely used for building insulation, where humidity has detrimental effects on PS insulation properties [24], and yet there are almost no reports of fundamental measurements quantifying water sorption in PS. This is perhaps due to the challenge of measuring small absolute quantities of equilibrium water uptake in PS.

Water is known to have low solubility in PS, but there are only a few reports of water sorption in PS [25,26,27]. Near room temperature, water solubility mass fractions are between 10^−4^ and 10^−3^ at water activities of 0.7 to 0.8 [25,26,27]. The temperature dependence of water solubility in PS within a given study is weaker than the variability between studies, which may be due to differences in molecular weight, and, as we will show, synthetic method. At such low solubilities, indeed, slight changes in the polymer structure, the presence of polar chain ends, and difference in the molecular weight (and its distribution) may play a significant role in the resulting water uptake at equilibrium. Furthermore, tacticity is known to have an effect on penetrant solubility. With different synthetic techniques the tacticity of PS can be controlled. The vast majority of PS in use is atactic. However, amorphous phases of syndiotactic PS present a larger free volume than the corresponding atactic polymer, so that it is characterized by a larger gas solubility [28,29]. Therefore, water sorption was measured with a manometric technique that we used previously to measure water sorption in polystyrene-*block*-poly(ethylene oxide) (SEO) BCP samples [30]. In SEO samples, water sorption is dominated by the PEO phase [30]. Sorption of good solvent vapor is known to plasticize glassy polymers such as PS [31,32]. Solubility and interaction of PS with many organic solvents is well known [33]. The most widely studied solvents in PS are toluene and benzene (good solvents) [34,35], cyclohexane (theta solvent at 34 °C) [36,37], and methylethylketone (marginal solvent) [38,39]. In most of these systems, the Flory-Huggins interaction parameter, χ, increases with increasing polymer volume fraction. The increase tends to be weak $\left(\chi \propto \phi_{p}^{1/2}\right)$ at low polymer fraction but much stronger at high polymer fractions [37], although there is indication that it actually decreases in the PS-toluene system [34]. This indicates that χ (a first order polymer-solvent interaction parameter) is not sufficient to capture the free energy of the system and that higher order terms are needed. This finding raises an interesting question: what occurs in the limit of pure polymer? Gas chromatography has been used to determine interaction parameters in the limit of pure PS for a wide range of solvents above PS *T_g_* [40]. Of those examined, water is the poorest solvent with an interaction parameter that ranges between 3 and 4.4 depending on temperature. Despite the pervasive presence of water vapor, its effect on PS *T_g_* has not been examined.

Generally, an external plasticizer is defined as a component that improves the properties of interest for a particular application, such as increasing flexibility, toughness, or elongation at break as well as decreasing processing temperature [41]. Polymer-polymer and polymer-plasticizer interactions must be of similar energy so that plasticizer molecules can screen polymer-polymer interactions. Therefore, to understand the effect of water on PS and its potential plasticization of different PS grades, it is important to quantify the surface energy of the resulting polymer obtained via different synthetic methods. For amorphous polymers, such as atactic PS, plasticization effect on *T_g_* can be quantified with thermal, mechanical, and dielectric techniques [41]. The depression of *T_g_* is one of the most significant and most frequently quantified effects of plasticization of polymers.

The *T_g_* of PS is commonly reported as about 100 °C [42], although, like most polymers, it is a function of molecular weight as well as tacticity. The effect of molecular weight on *T_g_* is accepted to be due to internal plasticization [41] by chain ends [43,44]. This effect saturates at high molecular weight, *M_n_*, as the concentration of chain ends become negligible according to the Flory-Fox equation [45]:

(1) $$T_{g}=T_{g,\infty }-\frac{A}{M_{n}}$$

The semi-empirical constant, *A* = 2*N_A_V_e_*/α, has been related to free volume of the chain ends, *V_e_*, and the difference between thermal expansion coefficients above and below *T_g_*, α [46]. *N_A_* is Avogadro’s constant. The glass transition temperature at infinite molecular weight, $T_{g,\infty }$, is a function of backbone chemistry and, presumably, backbone tacticity. This study will focus on the high molecular weight limit in which the value of *T_g_* plateaus to a constant value.

We have previously studied water sorption and transport in amphiphilic block copolymers of PS and poly(ethylene oxide) (PEO) that are of interest for membrane-based carbon dioxide capture and flue gas drying [30,47,48], in order to decrease power plant greenhouse gas emissions and improve energy efficiency [30,48]. During these studies it was discovered that water vapor impacts the *T_g_* of PS. The present article describes an investigation of this unexpected result in PS homopolymers of four different molecular weights and in block copolymers of two different morphologies. We present complementary techniques for measuring *T_g_* using differential scanning calorimetry (DSC) and dynamic mechanical analysis (DMA) as a function of water activity. The two techniques have reasonable agreement and show a notable decrease in *T_g_* with increasing water activity. Measuring the effect of water on the glass temperature of PS will potentially aid in modeling membrane performance in applications such as water filtration or desalination [49,50] and gas separation [47,51] as well as providing possible insight into plasticization of polymers.

## 2. Materials and Methods

### 2.1. Materials

The properties of PS homopolymers and block copolymers used in this study are reported in Table 1. The PS homopolymers are denoted PS#k, where # is the number-averaged molecular weight (*M_n_*) in kg/mol. The diblock copolymers composed of PS and PEO (SEO) are denoted SEO#k, where # is the *M_n_* of the PS block. The polydispersity index (PDI) is reported for the PS block of the block copolymers (BCPs). The PS volume fractions are reported at a reference temperature of 40 °C. The morphology of the BCPs was determined by small angle X-ray scattering, reported elsewhere [30,48]. All the polymers used in this study, except PS109k, were synthesized via anionic polymerization [52]. Of the four PS samples, PS77k and PS160k were used as-received from Polymer Source (P3911-S and P4250-S, respectively), where they were anionically synthesized at −78 °C in tetrahydrofuran. The PS387k homopolymer and the block copolymers were anionically synthesized in-house at room temperature in benzene, as reported previously [48]. PS109k is a peroxide-initiated polymer synthesized by free radical polymerization. It was kindly provided by Versalis and used without further purification. The molecular weights of the PS and polydispersity index were measured by gel permeation chromatography (Viscotek GPCmax, Malvern, UK with dimethylformamide solvent and 0.05 M LiBr). The volume fraction of the PS block in each copolymer was determined using ^1^H nuclear magnetic resonance (NMR) spectroscopy at 25 °C. For inverse gas chromatography measurements, the samples were used as received. For all other measurements, samples were formed into dense films via casting from *N*-methyl-2-pyrrolidone solution as described elsewhere [48], or from toluene, followed by annealing under vacuum at 120 °C for at least 3 h and slowly cooled to room temperature.

### 2.2. Water Sorption Measurements

Water sorption experiments were conducted in a fixed-volume pressure decay apparatus, which makes use of a manometric technique to evaluate the water uptake from the pressure change of the vapor phase during sorption in a calibrated volume, as described elsewhere [53,54].

Water uptake was obtained from the asymptotic pressure value of the vapor phase, from which the equilibrium water activity (*a_w_*) is also calculated. We define the standard state for sorption experiments as pure water vapor at the temperature of the experiment.

*a_w_* = *P/P^sat^* (2)

where *P* is water vapor pressure and *P^sat^* is the saturated vapor pressure at the test temperature. The rate of change of pressure was also used to determine the diffusion coefficient of water, as described previously [30]. Tests were performed by increasing *a_w_* stepwise up to 80% RH at temperatures between 35 and 75 °C. At each temperature, at least two consecutive sorption experiments have been carried out in all samples considered.

### 2.3. Dynamic Mechanical Analysis

Tensile oscillatory measurements were performed using a Q800 dynamic mechanical analyzer (DMA, TA Instruments, New Castle, DE, USA) equipped with an accessory that controls the relative humidity [55]. DMA experiments are in air and humidity is actively controlled. Thus, *a_w_* is well-defined, and the reference state is such that *a_w_* = 1.0 at 100% RH. Temperature ramp experiments were conducted at an oscillatory strain of 10 µm amplitude and a frequency of 1 Hz for storage and loss moduli measurements. The sample temperature was ramped from 60 to 110 °C at a rate of 1 °C/min.

### 2.4. Thermal Analysis

The *T_g_* of PS membranes were measured as a function of *a_w_* using differential scanning calorimetry (DSC, TA Q1100 with RCS event control, TA Instruments, New Castle, Delaware, USA). Dry samples were sealed in pans directly in an argon glove box without exposure to air. Each humidified sample was equilibrated for 12 h at room temperature (24 ± 1 °C) in a humidification chamber that controlled *a_w_*. In discussing DSC results we refer to this initial equilibration as the nominal water activity, *a_w,nom_*, recognizing that as the temperature of the sealed DSC pan is increased during a measurement, the actual water activity is changing according to:

(3) $$a_{w}=\frac{P_{w,nom}\cdot \;T}{P^{sat}\;\cdot \;T_{nom}}$$

At $T_{nom}=24\;{}^{\circ}C$, $P_{w,nom}=a_{w,nom}P_{nom}^{sat}$, where $P_{nom}^{sat}=29.82\;\mathrm{mbar}$. $P^{sat}$ is a function of temperature, and values were taken from saturated steam tables [56]. We are interested in the water activity at the PS *T_g_*, which ranged between 83 and 110 °C in this study. Key assumptions are (1) that the DSC pan is sufficiently large that the vapor phase condition is not significantly affected by sorption/desorption of water by the sample and (2) that equilibration occurs instantaneously between the DSC sample and the vapor phase. Specialty stainless steel pans (PerkinElmer, Waltham, MA, USA) were required to maintain a sealed environment. These pans are considerably larger than standard aluminum DSC pans, and the first assumption is most likely a good approximation. Samples were free-standing membranes with thicknesses ranging between 80 and 90 μm. The equilibration time can be estimated around 20 s ($t_{eq}\approx L^{2}/D$) for a 90 μm thick sample, based on a diffusion coefficient of water in PS at 356 K of $D=1\times 10^{-6}\;{\mathrm{cm}}^{2}/s$ [30]. At 383 K, $D=2\times 10^{-6}\;{\mathrm{cm}}^{2}/s$, and $t_{eq}=8\;s$, using an Arrhenius activation energy of 37 kJ/mol [30]. DSC scans were conducted between 60 °C and 140 °C at a scan rate of 5 °C/min. Thus, the actual water activity at PS *T_g_* is accurate to within 5/3 °C. In other words, the actual water activity accuracy is similar to the accuracy of the *T_g_* measurement itself. Repeated heating and cooling runs were performed, and average values reported for repeated runs on at least two samples for each humidity condition.

### 2.5. Surface Energy Analysis

All surface energy analyses were carried out using iGC Surface Energy Analyzer (SMS, Alperton, UK) and the data were analyzed using both standard and advanced SEA Analysis Software. The injection system used by the iGC SEA allows the precise control of the injection size, therefore different amount (mole, *n*) of probe vapor can be chosen to pass through the sample column to achieve different surface coverages, *n*/*n_m_*. If a series of probe vapors is injected at the same surface coverage, the surface energy and Gibbs specific free energy values can be determined. Consequently, the injections of probe vapors at different surface coverages will result in a distribution of surface energy as a function of surface coverage, which is referred as a surface energy profile. The determination of surface energy heterogeneity by iGC SEA can, therefore, be described as a mapping technique. Detailed methodology has been described elsewhere [57,58].

For all experiments, material was packed into individual silanized glass columns (300 mm long by 4 mm inner diameter) using the SMS Column Packing Accessory. Each column was conditioned for a period of 1 h at 30 °C and 0% RH with helium gas prior to any measurements. All experiments were conducted at 30 °C with 10 SCCM total flow rate of helium gas, using methane for dead volume corrections. Samples were run at a series of surface coverages with n-alkanes (nonane, octane, heptane and hexane; Aldrich, HPLC grade) and polar probe molecules (acetone, ethanol, acetonitrile, ethyl acetate, and dichloromethane; Aldrich, HPLC grade) to determine the dispersive surface energy as well as the specific free energies of adsorption, respectively. For the surface energy analysis, the method of Dorris and Gray was employed for the dispersive surface energy component. [59] For the specific free energies of desorption, the Polarization approach was employed [60]. Acid-base surface energy components were determined using the Good-van Oss-Chaudhury (GvOC) model [61,62] where the acid-base (*γ_s_^AB^*) component is taken as the geometric mean of the Lewis acid parameter (*γ_s_*^−^) and Lewis base parameter (*γ_s_^+^*).

## 3. Results

**Figure 1:**
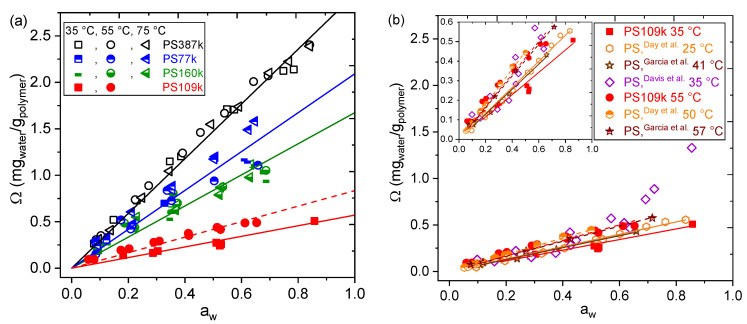
(**a**) Water sorption isotherms in PS normalized to total polymer mass at various temperatures. Lines are best fits of Equation (4) to each data set. All temperature measurements for each data set are included in the fits, with the exception of PS109k in which the solid line is fit to data at 35 °C and dashed line is fit to data at 55 °C. (**b**) Comparison of water sorption isotherms for PS109k with literature: Day et al. [26], Garcia et al. [25], and Davis et al. [27]. Solid lines are fits to each data set below 50 °C, whereas dashed lines are fits to each data set at or above 50 °C. Inset is the same data shown over a smaller vertical axis range for clarity.

The water sorption results for PS samples are reported in Figure 1a from 35 to 75 °C. The water uptake is expressed as mass ratio, Ω. In the custom anionically synthesized sample, PS387k, Ω is linear with *a_w_* and has a solubility coefficient, *k* = 2.9 ± 0.1 mg_water_/g_polymer_, that is independent of temperature within the range investigated. This solubility coefficient is defined according to:

Ω = *k*∙*a_w_* (4)

Interestingly, the other PS homopolymers take up less water than PS387k. Although the water sorption isotherms of PS77k and PS160k appear to have a possible slight temperature-dependence, there is not a clear trend with temperature. Therefore, the data sets at all three temperatures were included in the linear regression to determine solubility coefficient of each polymer. The regression results are shown in Figure 1a and reported in Table 2. Based on the 95% confidence interval reported in Table 2 and considering the small quantities of water being taken up (mg_water_/g_polymer_), neither the differences due to temperature nor those due to molecular weight appear to be statistically significant for PS77k and PS160k (both from Polymer Source). The variance of *k* mirrors that of the measurements themselves that have a standard deviation of approximately 10%. However, there is a real difference between water sorption in the Polymer Source samples (anionically synthesized at low temperature) and water sorption in PS387k (anionically synthesized in-house at room temperature). Most significantly, the commercial PS (PS109k) has lower Ω than any of the anionically synthesized PS samples. As with the other PS homopolymers, the water sorption isotherm is linear with *a_w_*. As shown in Figure 1b, the results for the commercial PS109k compare well with several reports from literature. The significant difference in the amount of water sorbed by PS109k and PS387k is corroborated by surface energy measurements with inverse gas chromatography (Figure S1) in which PS387k exhibits significantly greater wettability than PS109k. The presence of hydrophilic chain ends (Table 2) with hydroxyl functionality is indeed expected to provide such an effect.

In order to evaluate the differences in water sorption with synthesis method, aiming to identify the role of the chain ends in determining the resulting water solubility, the number of water molecules per chain end was calculated according to the following:

(5) $$\Omega^{*}=\frac{\Omega /M_{w}}{2/M_{n}}$$

Correspondingly, k* is the solubility coefficient from the linear correlation of $\Omega^{*}$ with water activity. The molar mass of water, $M_{w}=18$ g/mol, and the number-average molar mass of each polymer as reported in the naming convention was used for $M_{n}$. The 2 in the denominator treats the polymers as linear and the chain ends as identical. The error of $\Omega^{*}$ could be as much as a factor of 2 due to this approximation. For peroxide initiated PS, such as PS109k from Versalis, it has been shown that the number of benzoate carbonyl functional groups per chain is between 1.4 and 2.3 [63], such that this approximation is of the correct order of magnitude for PS109k. Of course, we are not proposing that water molecules reside *only* at chain ends since there is presumably free volume throughout the glassy polymer available for water molecules to occupy, but we are using $\Omega^{*}$ as a metric to evaluate the significant difference in amount of water sorbed by the differently synthesized PS samples, because the most significant different in synthetic methods is expected to be the chemistry of chain ends. Regarding the other anionically synthesized polymers, one chain end is a butyl group and the other chain end is either a hydrogen (in the case of the Polymer Source samples, PS77k and PS 160k) or a hydroxyl (in the case of the custom PS387k). The hydroxyl chain end is obtained by termination with ethylene oxide followed by termination with degassed methanol. Considering the apparent hydrophobicity of the chain ends of PS77k and PS160k, it is unexpected that they take up more water than PS 109k. This could indicate that PS77k and PS160k contain more free volume than PS109k due, for instance, to a difference in tacticity. Low anionic synthesis temperature is known to favor syndiotacticity, [64] which in turn is characterized by a larger free volume. The value of $\Omega^{*}$ for PS387k is much greater than that of PS109k, which is expected for hydrophilic chain ends such as hydroxyl. However, the number of water molecules per hydroxyl group in PS387k is considerably larger than what might be expected based on water sorption in poly(vinyl alcohol) (PVA). Using reported results for liquid water sorption in PVA [65], we find a value of approximately 6 water molecules per vinyl alcohol monomer. PS387k has an order of magnitude larger chain-end-normalized water uptake, much larger than the difference between Polymer Source and Versalis samples. Thus, free-volume alone cannot account for the order of magnitude increase in $\Omega^{*}$ of PS387k, the most likely explanation is that hydroxyl chain ends form hydrophilic domains that can accommodate clusters of water. In summary, there are two main effects at play. One, syndiotactic PS has greater free volume and thus sorbs more water than atactic PS. Two, PS with hydrophilic chain-end functionality has significantly greater water sorption than PS with hydrophobic chain ends.

**Figure 2:**
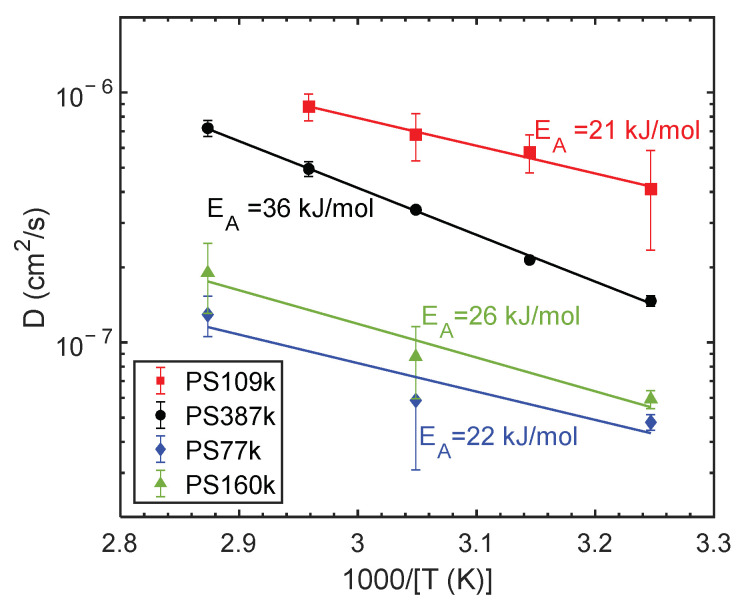
Arrhenius plot of water diffusion coefficients in different PS samples denoted in the legend. Solid lines are Arrhenius fits to the data with activation energy noted. Diffusion coefficients at each temperature are averaged over all water activities due to lack of dependence on water activity. Error bars represent one standard deviation.

Water diffusion coefficients were also determined from sorption measurements by fitting the transient response, according to the solution of the Fick’s law for the experimental conditions provided by Crank [66]. The complete data set is reported in the supporting information. The Arrhenius temperature dependence of water diffusion coefficient in the different grades of PS inspected is reported in Figure 2. The diffusion coefficient does not show any apparent trend with water activity (see Figure S2), as expected in view of the low solubility. So, the diffusion coefficients at each temperature are mean values of those calculated in all isothermal sorption steps. As one can see, the diffusion coefficient of the different PS samples spans over about one order of magnitude, and D_109k_ > D_387k_ > D_160k_ > D_77k_. Interestingly, the two anionically synthesized PS samples are those presenting the lowest water diffusivity, while the largest diffusivity is observed for the Versalis grade 109k, which is the one with the lowest solubility. Such difference between the commercial PS109k and the more highly syndiotactic PS77k and PS160k further supports the assertion that the differences in water sorption are due to an interplay between free volume distributions and the effect of polar chain-ends. The diffusion coefficient values also indicate that thermodynamic effects, e.g., reFiglated to custom PS387k chain ends, are primarily responsible for the large amount of water sorbed by PS387k. If free volume differences were the dominant cause of differences between PS387k and PS109k, then the water diffusion coefficients would be expected to be much lower than results indicate. The activation energies, *E_A_*, are comparable for the 4 different grades, ranging from 21 kJ/mol for the Versalis grade (PS109k) to 36 kJ/mol for PS387k with hydrophilic chain ends. Interestingly, the larger temperature variation of diffusivity, and thus the larger *E*_A_, is observed for the hydroxyl-terminated anionically synthetized PS387k grade, which in turn is the one that shows the most favorable interaction with water and larger solubility (Figure 1). Resulting *E_A_* for the other 3 grades are similar to each other (21–26 kJ/mol). In summary, water diffusion in all samples had an Arrhenius temperature dependence and no apparent water activity dependence. Complete data sets of all sorption and diffusion measurements are available in the supporting information data and Figure S2.

**Figure 3:**
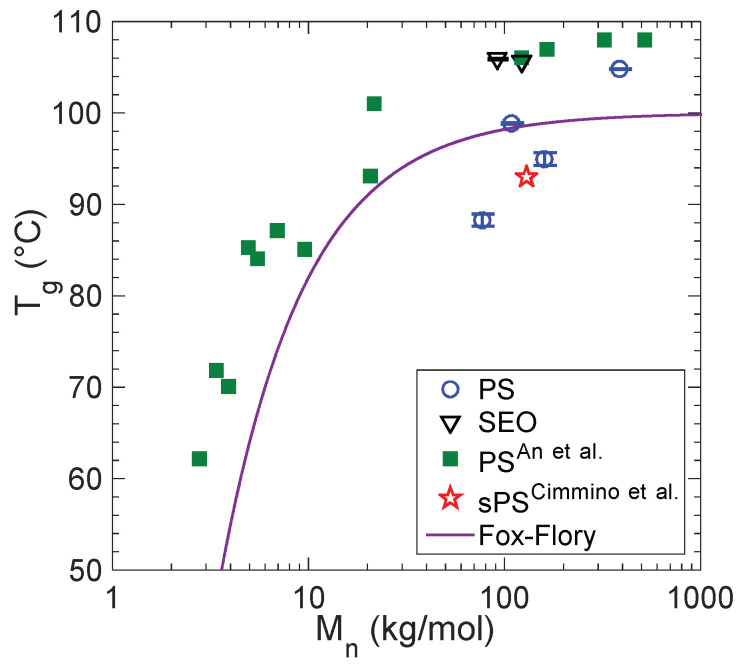
Comparing the PS *T_g_* of homopolymers (PS) and block compolymers (SEO) with literature: for narrow dispersity PS by An et al. [68], for syndiotactic PS by Cimmino et al. [72], and the Fox-Flory model applied to thermally polymerized and fractionated PS [70].

In order to examine other differences among the samples, the *T_g_* of PS homopolymers and the PS phase in SEO BCPs at different water activities were characterized. Figure S3 in the supporting information shows characteristic thermograms recorded during the second heating scan for the case of PS160k. For all polymers investigated, the fictive temperature, determined from heating scans, and the *T_g_*, determined from cooling scans, agreed to within 2.5 °C. Agreement is expected due to our using the same heating and cooling rates [67]. We focus on *T_g_* measurements and report the value at the inflection point. The *T_g_* of the PS homopolymers and the PS phase of the SEO BCPs in the dry state are reported in Figure 3 as a function of PS *M_n_*. Within experimental error, the SEO values agree with a literature report of PS with narrow molecular weight distribution also shown in Figure 3 [68]. PS *T_g_* values reach a high molecular weight limit at about 500 kg/mol where the *T_g_* plateaus at approximately 108 °C [69]. PS387k has slightly lower *T_g_* than the narrow-molecular-weight-distribution literature comparison. The *T_g_* of the commercial PS109k agrees well with the Fox-Flory equation that was based on data from thermally polymerized and fractionated PS [70].

As shown in Table S1, the solvent from which PS109k films are cast has no effect on the *T_g_*. It also agrees with DSC measurements of the same polymer taken on a different instrument (*T_g_* = 99.9 °C) [71]. The PS160k and PS77k have much lower *T_g_* than is expected based on molecular weight and polydispersity differences. The observed differences in glass transition may be ascribed to variation in polymer tacticity of the different grades of PS, as mentioned above. Syndiotactic PS (sPS) indeed has significantly lower *T_g_* than the more widely studied atactic PS. In fact, the *T_g_* of 129 kg/mol fully syndiotactic PS is 93 °C [72], in good agreement with our results for the Polymer Source PS samples. It is worth noting that although PS77k and PS160k appear to have some syndiotacticity, it is not sufficiently high to detect crystallinity with DSC.

**Figure 4:**
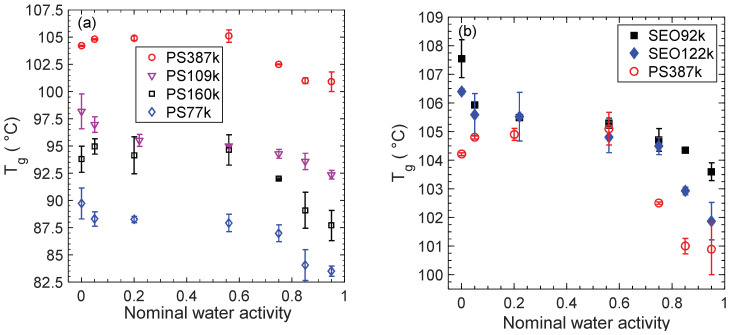
Effect of water activity on the *T_g_* of (**a**) the PS samples and (**b**) the PS phase in SEO samples with PS387k shown for reference.

The effect of water activity on the *T_g_* of PS is presented in Figure 4a for the homopolymers and in Figure 4b for the SEO BCPs. PS387k is also shown in Figure 4b for reference. There appears to be some variability of *T_g_* at low nominal *a_w_*, but it is not statistically significant for most of the PS samples in Figure 4a. In Figure 4b, at a nominal *a_w_* of 0.05 the *T_g_* of the PS phase of SEO92k is 105 ± 1 °C, somewhat lower than that of the dry membrane in argon. PS387k homopolymer *T_g_* is 104.8 ± 0.1 °C at 0.05 water activity, slightly higher than that of the dry membrane in argon. Argon at 1 atm has an order-of-magnitude lower solubility in PS than that of water (10^−4^ by mass fraction) [73], and the effect on *T_g_* is minor at most. The most likely explanation is that the DSC measurement of dry PS is adversely affected by the order of magnitude lower thermal conductivity of argon as compared to air. The lowest achievable humidity in air, i.e., the humidity-controlled chamber’s lower limit, is 5% RH. Therefore, *T_g_* of all samples at a nominal *a_w_* of 0.05 are reported in the second column of Table 3.

For each polymer there is little change in *T_g_* up to a nominal *a_w_* of 0.56, or an actual *a_w_* of about 0.025. At nominal *a_w_* = 0.75, there is an onset of *T_g_* depression in the PS homopolymers, as shown in Figure 4a. For example, PS387k *T_g_* decreases by 2.6 °C from *a_w_* = 0.56 to *a_w_* = 0.75. For the BCPs, this onset of *T_g_* depression occurs at higher nominal *a_w_* = 0.85. For example, the PS *T_g_* of SEO122k decreases 1.6 °C from *a_w_* = 0.75 to *a_w_* = 0.85. The total depression of *T_g_* (noted Δ*T_g,max_* in Table 3) was calculated between 5% RH and 95% RH (the minimum and maximum relative humidity values at which DSC samples were equilibrated in air). Both PS homopolymers and PS-containing BCPs follow a similar trend. The decrease in PS *T_g_* from the dry state (5% RH) to the highest humidity (95% RH) is statistically significant with an average decrease for all the samples of 4.4 ± 0.4 °C. An interesting aside, is that the SEO samples, for the most part, have higher *T_g_* values than PS387k despite having lower PS M_n_. Such behavior could be due to nanoconfinement in block copolymer structure, which has previously been shown to affect *T_g_*, albeit in much thinner films [74].

**Figure 5:**
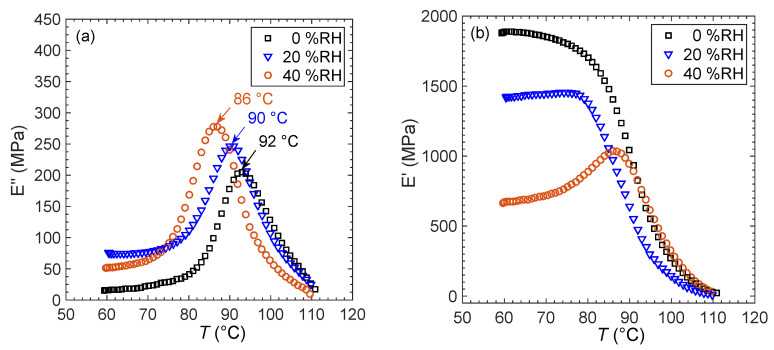
(**a**) Determination of PS160k *Tg* from loss modulus measurement at controlled relative humidity. (**b**) Effect of relative humidity on the tensile storage modulus of PS160k.

In order to corroborate DSC results, humidity-controlled dynamic mechanical analysis was used to determine the *T_g_* of PS160k. The results are shown Figure 5a, where *T_g_* was taken as the maximum of the tensile loss modulus, *E*″. We follow the approach of Rieger [75] in this regard, who describes why defining the *T_g_* at the maximum of the loss modulus is superior to using the maximum of tan δ. As the water vapor concentration is increased from 0% RH to 40% RH the peak of the loss modulus (indicative of *T_g_*) decreases. A direct comparison of the *T_g_* of PS160k in the dry state (0% RH) measured by DSC (94 ± 1 °C) with that measured by DMA (92 °C) indicates minor disagreement between the two techniques. However, both techniques show a similar trend of decreasing *T_g_* with increasing water activity. The magnitude of the decrease in *T_g_* (DSC = 4.4 ± 0.4 °C, DMA = 6 °C) is significantly greater than the 2 °C discrepancy between the two techniques. Unfortunately, samples with *T_g_* above 100 °C could not be investigated with humidity-controlled DMA due to limitations of the instrument.

Figure 5b shows the temperature dependence of the tensile storage modulus for the PS160k membrane at three different relative humidities. The elastic modulus, *E*’, in the dry state (0% RH) is 1.88 GPa at 60 °C. This value compares reasonably well with literature reports of *E*’ for PS in the glassy state, which is 2.9 ± 0.6 GPa [33,42,76,77,78,79]. When the humidity of the surrounding environment is controlled at 20% RH, *E*’ below *T_g_* is about 500 MPa less than in the dry state. At 40% RH and 60 °C, *E*’ is about one-third of the dry value. We do not have a definitive explanation for the increase in elastic modulus as *T_g_* is approached at 40% RH. This could be due to water sorption decreasing with increasing temperature, but such behavior is not supported by our water sorption results within the range of temperatures accessible in our sorption experiments. Isothermal solute sorption has been shown to increase polymer modulus in other systems by filling excess free volume [80,81], and excess free volume is expected to decrease as *T_g_* is approached from below [82], which could potentially explain this result. However, definitive explanation for the non-monotonic mechanical response of PS in the presence of water requires detailed computational simulations. Above *T_g_*, the elastic modulus of PS160k drops to a small value as it becomes rubbery. This drop is for the most part independent of water activity, suggesting that, above the glass transition, the effect of water on PS segmental mobility is small.

**Figure 6:**
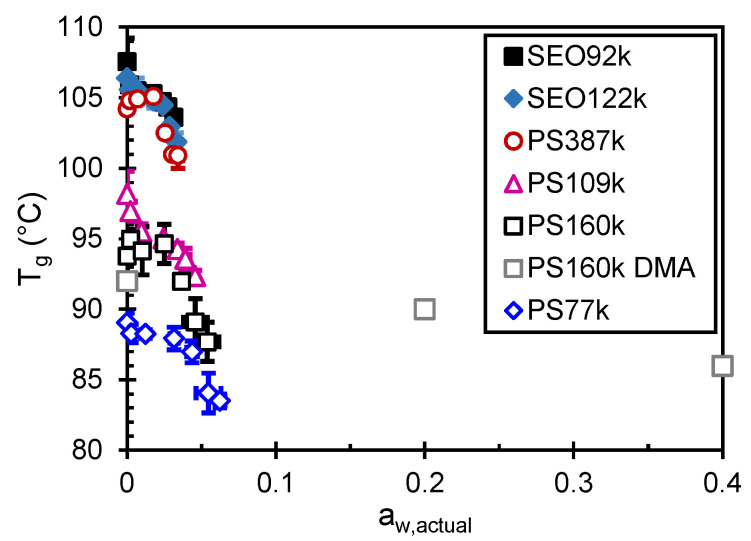
Effect of (actual) water activity on the *T_g_* of the PS samples and PS phase in SEO samples, comparison of the DSC results with those obtained by DMA.

Humidity-controlled measurements of *T_g_* are experimentally challenging. For example, DSC experiments rely on equilibrating samples at room temperature and a nominal water activity. DSC measurements are then made as a function of temperature in a closed system. Due to phase equilibrium between the sample and headspace in the DSC pan, we do not accurately know the water activity at the measured *T_g_*. Note that the temperature is scanned at 5 °C/min in DSC experiments and the water diffusion time is on the order of 1 min, such that it is not clear that equilibrium water activity nor equilibrium water sorption is exactly achieved at *T_g_*. On the other hand, the humidity during DMA experiments is actively controlled at atmospheric pressure, so that water activity is accurately known at *T_g_*. However, this limits measurements to temperatures below approximately 100 °C and low humidities. The absolute results of DSC and DMA are not readily compared in the humidified state for this reason. An attempt to do so is shown in Figure 6. *T_g_* from DSC are plotted versus the actual *a_w_*. It is important to emphasize that *a_w_* is known accurately in DMA experiments, more reliably than the estimations provided by Equation (3) for DSC.

The effect of sorbed water molecules on the PS glass transition is clear, although DMA and DSC results report quite different trends with water activity, due to the aforementioned reasons. It is striking, however, to note that even such small amount of low molecular weight species is able to produce apparent differences in the polymer *T_g_*. See Supporting Information, especially Figure S4, for details. Although the solubility of water in PS is low, it is one to two orders of magnitude greater than the solubility of water in polyethylene, which is 20 ppm on a weight basis [83]. This is due to specific interactions between water protons and pi electrons of the phenyl group. Spectroscopy and ab initio calculations of water-benzene have determined the binding energy of this interaction to be between that of van der Waals attraction and that of hydrogen bonding [84]. Such interactions are likely to produce the observed behaviors on glass transition depression, even if the actual amount of water sorbed is small.

## 4. Conclusions

In conclusion, we have shown that water sorption in a hydrophobic polymer such as polystyrene can be significantly affected by the synthesis method. The chain end functionality, combined with the extent of tacticity and free volume distribution are expected to be responsible for a 5-fold difference in water solubility from a commercial free-radical polymerized PS109k (low sorption) to an anionically synthesized PS387k (larger solubility). Differences have been observed also on the *T_g_* behavior of PS, which was found to decrease in the presence of water vapor. PS *T_g_* was measured with two complementary techniques, DSC and DMA. The error in estimating water content in DSC samples is quite large. Thus, DMA results are more reliable. *T_g_* values measured by DSC and DMA agree reasonably well. Due to water being a poor solvent for PS, measurements approaching the limit of pure polymer were possible, which fill a gap in the range of solvent fractions investigated for *T_g_* depression. Another significant finding was the surprising magnitude that the tensile storage modulus (measured at 1 Hz) decreases with increasing water activity. This could have consequences on the performance of membranes containing PS that are exposed to water or humid environments.

## Figures and Tables

**Table 1:** Properties of polystyrene (PS) homopolymers and PS-*b*-poly(ethylene oxide) (SEO) block copolymers used in this study: number-averaged molecular weight (*M_n_*), polydispersity index (PDI), and volume fraction.

	Supplier/Morphology	*M_n_* kg/mol	PS PDI	PS Volume Fraction (φ)
PS77k	Polymer Source	77	1.05	1
PS109k	Versalis	109	2.64	1
PS160k	Polymer Source	160	1.05	1
PS387k	In-house	387	1.02 ^a^	1
SEO92k	In-house/Cylindrical (PEO cylinders)	92	1.01	0.7
SEO122k	In-house/Lamellar (PEO majority)	122	1.02	0.46

^a^ High molecular weight fraction (800 kg/mol, 12% of total) excluded.

**Table 2:** Solubility coefficients from linear regressions to water sorption isotherms with intercept fixed to zero.

Sample	*k* (mg_water_/g_polymer_)	k* (mol_water_/mol_chain end_)	Chain End Functionality
PS387k	2.9 ± 0.1	31	Hydroxyl
PS77k	2.1 ± 0.2	4	Alkyl
PS160k	1.7 ± 0.2	8	Alkyl
PS109k (35 °C)	0.55 ± 0.03	2	Benzoate carbonyl
PS109k (55 °C)	0.83 ± 0.03	3	Benzoate carbonyl

**Table 3:** Results of *T_g_* measurements and subsequent analysis.

Sample	*T_g_* (5% RH) (°C)	Δ*T_g,max_* ^b^ (°C)
PS77k	88.3 ± 0.7	4.8 ± 0.8
PS109k	97.0 ± 0.7	4.6 ± 0.8
PS160k_DSC	95.0 ± 0.7	7.3 ± 1.5
PS160k_DMA	92 ^a^	6.0
PS387k	104.8 ± 0.1	3.9 ± 0.9
SEO92k	105.9 ± 0.1	2.3 ± 0.3
SEO122k	105.6 ± 0.7	3.7 ± 1.0
	All PS	5.1 ± 0.5
	All Samples	4.4 ± 0.4

^a^ Measured at 0% RH in air. ^b^ For DSC results, Δ*T_g,max_* is the difference between *T_g_* of samples equilibrated at 5% RH and those equilibrated at 95% RH. Equilibration was at 24 °C. For DMA, Δ*T_g,max_* is the difference between *T_g_* at 0% RH and 40% RH.

## Supplementary Materials

The following supporting information can be downloaded at: https://www.mdpi.com/article/10.3390/membranes12111059/s1, Figure S1: iGC Results; Figure S2: Diffusion Coefficients; Figure S3: DSC Thermograms; Table S1: Solvent Effect on *T_g_*; Table S2: Physical Constants for Prediction of *T_g_* Depression; Figure S4: Prediction-Experiment Comparison with Literature. References [32,85,86,87,88,89,90,91] are cited in the supplementary materials.
