# Supplementary material for: Effect of Polystyrene Synthesis Method on Water Sorption and Glass Transition
Source: Membranes (Basel). 2022 Oct 28;12(11):1059. doi: 10.3390/membranes12111059 (PMC9692681; doi:10.3390/membranes12111059)
Supplement: Supplementary file 1 [file membranes-12-01059-s001.zip › SI_2022-10-27.pdf]

Supporting Information for

Effect of Polystyrene Synthesis Method on Water Sorption and Glass Transition

Daniel T. Hallinan Jr.,[1,†,*] Matteo Minelli,[2,3,†,*] Onyekachi Oparaji,[1] Andrea Sardano,[3] Oluwagbenga Iyiola,[1] Armando R. Garcia,[4] Daniel J. Burnett [4]

[1a] Department of Chemical and Biomedical Engineering, Florida A&M University –- Florida State University College of Engineering, AME Center and Department of Chemical and Biomedical Engineering, 2525 Pottsdamer Street, Tallahassee, FL 32310, USA

[1b] Aero-propulsion, Mechatronics, and Energy (AME) Center, Florida A&M University–Florida State University College of Engineering, 2003 Levy Avenue, Tallahassee, FL 32310, USA

[2] Department of Civil, Chemical, Environmental and Materials Engineering (DICAM)— Alma Mater Studiorum, University of Bologna, via Terracini 28, Bologna , 40131, Italy

[3] Interdepartmental Center for Industrial Research in Advanced Mechanical Engineering Applications and Materials Technology (MAM)— Alma Mater Studiorum, viale del Risorgimento 2, Bologna, 40136, Italy

[4] Surface Measurement Systems, 2125 28th Street SW, Suite 1, Allentown, PA 18103, USA

[*] Correspondence: dhallinan@eng.famu.fsu.edu (D.T.H.J.); matteo.minelli@unibo.it (M.M.)

[†] These authors contributed equally to this work.

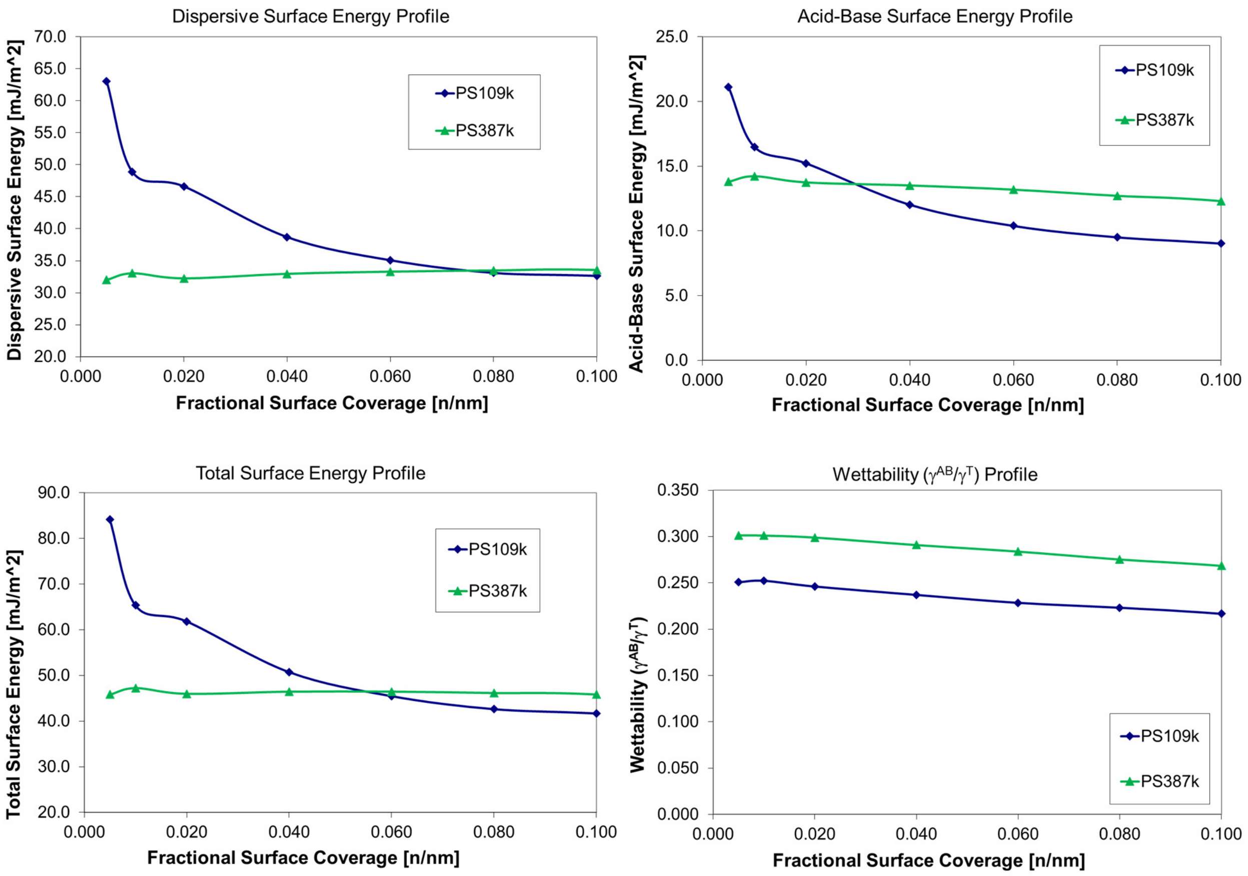

Figure S1. Inverse gas chromatography results for n-alkanes (top left) polar vapors (top right) and total surface energy profile (bottom left) as well as wettability (bottom right). The lack of dependence on fractional surface coverage exhibited by PS387k is indicative of a homogeneous surface profile in which all adsorption sites are of similar energy. The more heterogeneous surface exhibited by PS109k could be due to it having much larger dispersity. The wettability results support the sorption results; both indicate that the custom PS387k is a more hydrophilic polymer than the commercial PS109k.

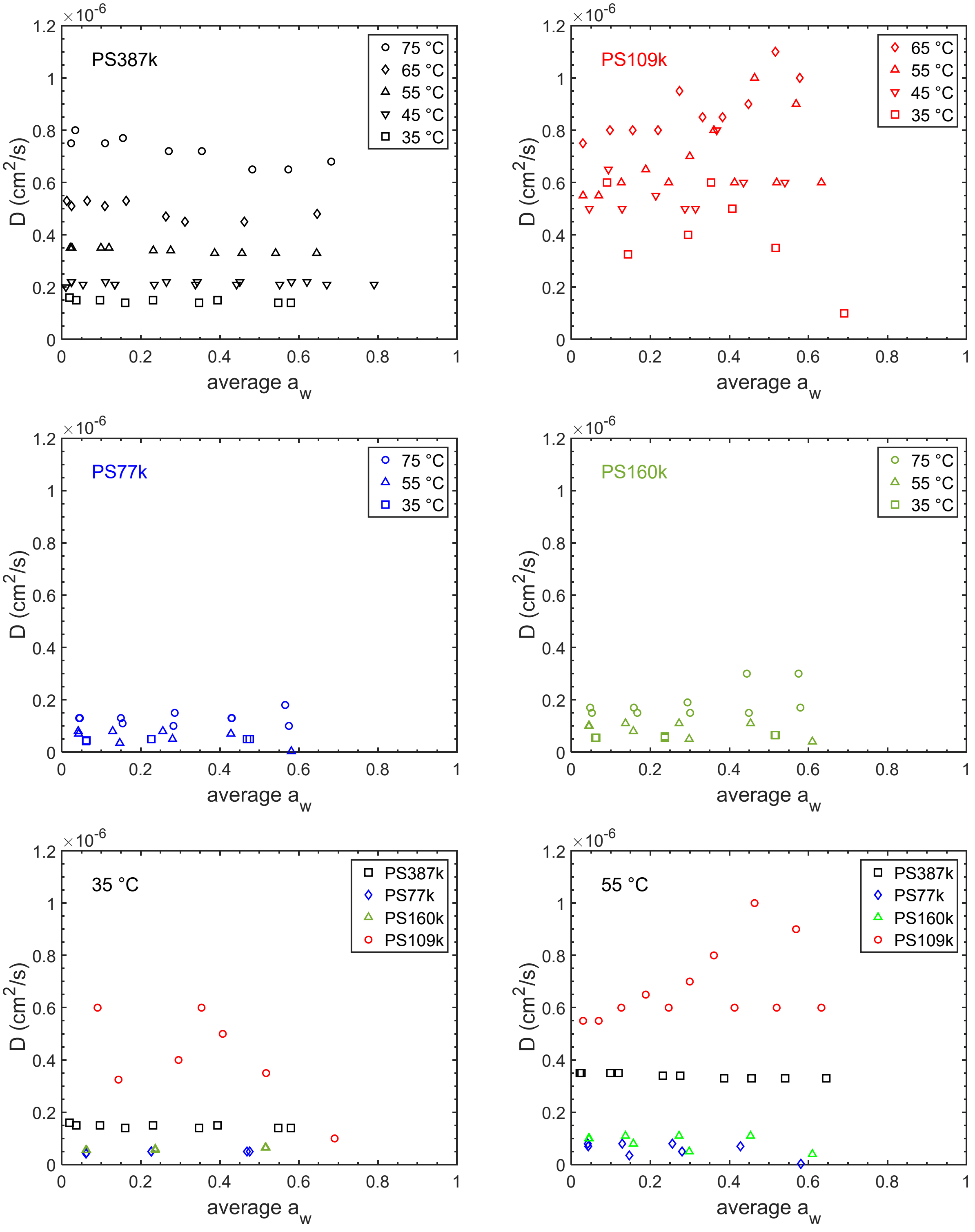

Figure S2. Plots of the water diffusion coefficient, D, from fits to transient water sorption data as a function of the average water activity, average a_w, of the measurement for the four grades of PS investigated in this study, at temperatures ranging from 35 °C to 75 °C.

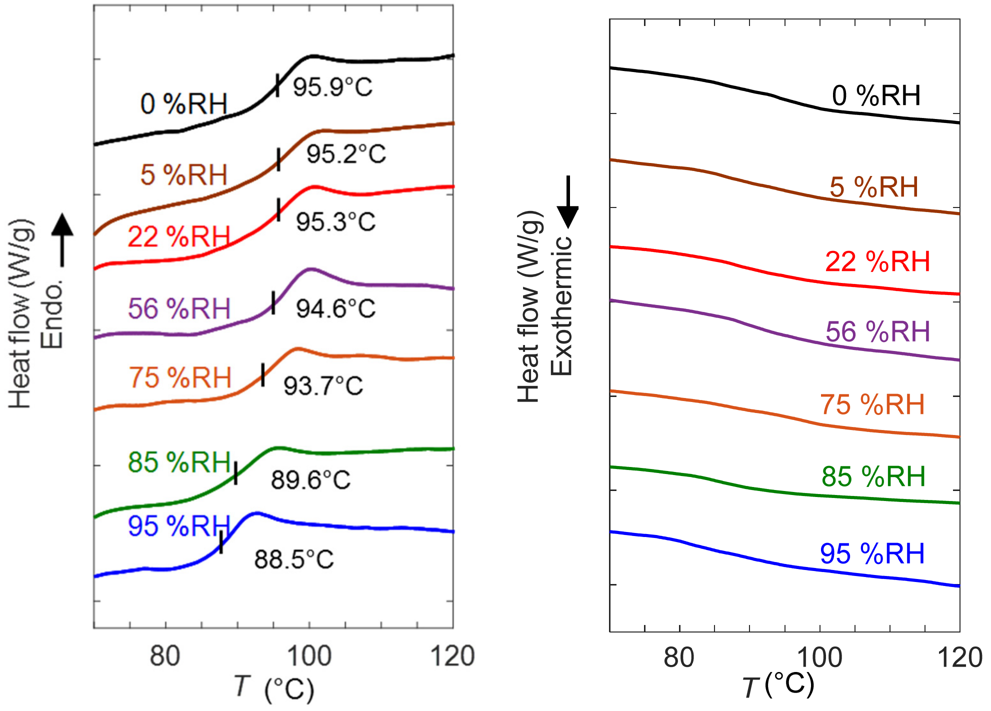

Figure S3. DSC thermograms of PS160k as a function of water activity for heating (left) and cooling (right). Each curve was taken at a rate of 5 °C/min and is offset for clarity.

Table S1. Effect of casting solvent on PS109k T_g, as measured by DSC.

	PS109k (Toulene)		PS109k (NMP)	
a_w	T_g (°C)	error	T_g (°C)	error
0	98.86	0.09	98.19	1.60
0.05	97.50	0.75	96.97	0.72
0.22	95.93	0.68	95.52	0.55
0.56	94.57	0.30	95.01	0.05
0.75	95.06	0.95	94.28	0.41
0.85	93.74	0.18	93.58	0.75
0.95	92.74	0.18	92.36	0.40

Theoretical predictions based on thermodynamics have been derived for plasticizer-based T_g depression that require only composition, T_g of each component, and the heat capacity difference between liquid and glassy states (at T_g for each component, denoted $\Delta C_{P,i}$).[1] In the limit of small mole fraction of diluent, x_2, ten Brinke, Karasz, and Ellis predict the slope of T_g versus x_2, where x_2 is the mole fraction of plasticizer.[1]

$$\frac{dT_g}{dx_2} = -\frac{\Delta C_{P,2}}{\Delta C_{P,1}}\left(T_{g,1} - T_{g,2}\right) \qquad \text{(S1)}$$

This assumes the absence of specific interactions and phase separation, which can increase or decrease the degree of plasticization, respectively.[1] When specific interactions such as hydrogen bonding are present, the linear prediction of T_g on composition can breakdown.[2] This has been observed for PS and toluene,[3] where presumably pi-pi interactions are at play. It has also been observed in PS and triphenylmethylchloride (TPMC), where it was suggested that the "cusp" where the discontinuous slope occurs is related to the onset of immiscibility.[4] Kovacs has described it theoretically as the point at which the excess free volume of the polymer goes to zero.[5] In theta solvents, such as cyclohexane, liquid-liquid coexistence has been observed in PS below the theta temperature. This causes T_g depression to cease with increasing solvent volume fraction. Thus, interesting behavior has been observed in both good and theta solvents, but in the limit of pure polymer the thermodynamic prediction of ten Brinke and coauthors holds true.[1] A poor diluent system such as PS/water is of particular interest because the magnitude of poor diluent that sorbs into a polymer at equilibrium is much less than good solvent. Thus, data in the limit of pure polymer are readily accessible to examine the universality of the thermodynamic prediction.

The model used here is based on mole fractions, x_i, where styrene monomers are considered component 1 and diluent (water) is considered component 2. Water uptake can be converted to molar ratio, $X = M_{styrene}\Omega/M_{H2O}$, using the molar mass of each component ($M_{styrene}$ = 104 g/mol and M_{H2O} = 18 g/mol). Then water mole fraction is

$$x_2 = \frac{X}{1+X}. \qquad \text{(S2)}$$

The most reliable slope (dT_g/dx_2) is from DMA for PS160k. This value, -2080 °C/(mol_{H2O}/mol_{Total}), compares reasonably well with the prediction of ten Brinke and coworkers, dT_g/dx_2 = -1748 K.[1] The experimental result was calculated from the DMA data of PS160k at 20 %RH and 40 %RH. The prediction was calculated using the values reported in Table S2.

Table S2. Physical constants reported by ten Brinke and coworkers and used to predict T_g depression.[1]

Component	ΔC_p (J/g K)	T_g (K)
(1) PS	0.27	380
(2) H_2O	1.94	134

To put our results in the context of literature, Figure S4 presents our results along with several results from literature in which T_g and ΔC_p of both components were reported. In this figure a normalized T_g depression is reported, where

$$\Delta T_{\mathrm{g,expt}} = \frac{T_{\mathrm{g,1}} - T_{\mathrm{g,mix}}}{T_{\mathrm{g,1}} - T_{\mathrm{g,2}}} \quad \text{and} \qquad \text{(S3)}$$

$$\Delta T_{\mathrm{g,calc}} = \frac{x_2 \Delta C_{\mathrm{p,2}}}{(1-x_2)\Delta C_{\mathrm{p,1}} + x_2 \Delta C_{\mathrm{p,2}}}. \qquad \text{(S4)}$$

When the glass transition temperature of the mixture, $T_{g,\mathrm{mix}}$, approaches that of the polymer, $T_{g,1}$, $\Delta T_{g,\mathrm{expt}}$ approaches zero. This tends to occur at low solvent contents. Conversely, when $T_{g,\mathrm{mix}}$ approaches that of

the solvent, $T_{g,2}$, $\Delta T_{g,\text{expt}}$ approaches one. This occurs at high solvent content. First, it can be seen that significant deviations from the theory occur above approximately 0.2. Second, the PS160k/water data from the current study fill a gap at the lowest values in Figure S4.

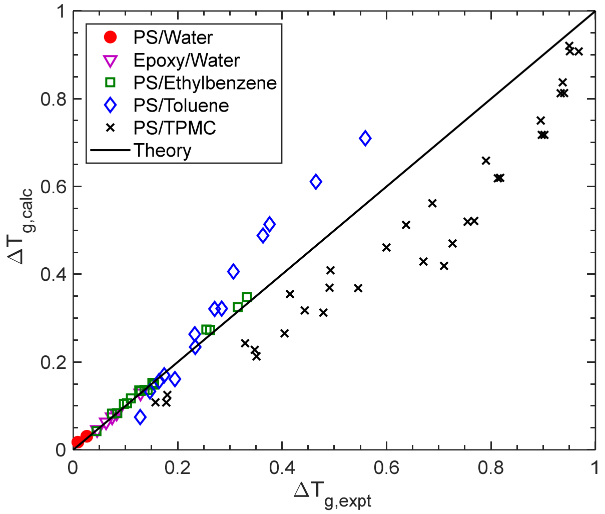

Figure S4. Comparison of predicted T_g depression, $\Delta T_{g,\text{calc}}$, and measured T_g depression, $\Delta T_{g,\text{expt}}$, for systems reported in literature in which the heat capacity difference is available: PS/Water,[This study] Epoxy/Water,[6] PS/Ethylbenzene,[7] PS/Toluene,[8] PS/TPMC (triphenylmethylchloride).[4]

Supporting Information References

1. ten Brinke, G.; Karasz, F.E.; Ellis, T.S. Depression of Glass-Transition Temperatures of Polymer Networks by Diluents. *Macromolecules* **1983**, *16*, 244-249, doi:10.1021/ma00236a017.
2. Kwei, T.K. The effect of hydrogen bonding on the glass transition temperatures of polymer mixtures. *Journal of Polymer Science: Polymer Letters Edition* **1984**, *22*, 307-313, doi:10.1002/pol.1984.130220603.
3. Braun, G.; Kovacs, A.J. Variations de la Température de Transition Vitreuse dans les Systèmes Binaires à Répartition Statistique. In Proceedings of the Physics of non-crystalline solids: Proceedings of the international conference, Delft, 1964; pp. 303-319.
4. Guan, L.; Sheng, J.; Huang, D. Investigation on Anomalous Calorimetric Behaviors of Polystyrene and Triphenylmethyl Chloride Blends. *Journal of Macromolecular Science, Part B* **2011**, *50*, 652-664, doi:10.1080/00222341003784832.
5. Kovacs, A.J. Transition vitreuse dans les polymères amorphes. Etude phénoménologique. Berlin, Heidelberg, 1964; pp. 394-507.
6. Moy, P.; Karasz, F.E. The Interactions of Water with Epoxy Resins. In *Water in Polymers*; ACS Symposium Series; American Chemical Society: 1980; Volume 127, pp. 505-513.
7. Ellis, T.S.; Karasz, F.E.; Brinke, G.T. The influence of thermal properties on the glass transition temperature in styrene/divinylbenzene network–diluent systems. *Journal of Applied Polymer Science* **1983**, *28*, 23-32, doi:10.1002/app.1983.070280103.
8. Bercea, M.; Wolf, B.A. Vitrification of polymer solutions as a function of solvent quality, analyzed via vapor pressures. *Journal of Chemical Physics* **2006**, *124*, doi:10.1063/1.2193153.
